# Methods to estimate changes in soil water for phenotyping root activity in the field

**DOI:** 10.1007/s11104-016-3161-1

**Published:** 2017-01-12

**Authors:** W.R. Whalley, A. Binley, C.W. Watts, P. Shanahan, I.C. Dodd, E.S. Ober, R.W. Ashton, C.P. Webster, R.P. White, M. J. Hawkesford

**Affiliations:** 1 Rothamsted Research, Harpenden, AL5 2JQ UK; 2 Lancaster Environment Centre, Lancaster University, Lancaster, LA1 4YQ UK; 3 NIAB, Huntingdon Road, Cambridge, CB3 0LE UK

**Keywords:** Phenotyping, Roots, Soil water profiles, ERT, EMI, Penetrometer

## Abstract

**Background and aims:**

There is an urgent need to develop new high throughput approaches to phenotype roots in the field. Excavating roots to make direct measurements is labour intensive. An alternative to excavation is to measure soil drying profiles and to infer root activity.

**Methods:**

We grew 23 lines of wheat in 2013, 2014 and 2015. In each year we estimated soil water profiles with electrical resistance tomography (ERT), electromagnetic inductance (EMI), penetrometer measurements and measurements of soil water content. We determined the relationships between the measured variable and soil water content and matric potential.

**Results:**

We found that ERT and penetrometer measurements were closely related to soil matric potential and produced the best discrimination between wheat lines. We found genotypic differences in depth of water uptake in soil water profiles and in the extent of surface drying.

**Conclusions:**

Penetrometer measurements can provide a reliable approach to comparing soil drying profiles by different wheat lines, and genotypic rankings are repeatable across years. EMI, which is more sensitive to soil water content than matric potential, and is less effective in drier soils than the penetrometer or ERT, nevertheless can be used to rapidly screen large populations for differences in root activity.

**Electronic supplementary material:**

The online version of this article (doi:10.1007/s11104-016-3161-1) contains supplementary material, which is available to authorized users.

## Introduction

Two of the most important functions of the root systems in crop species are to acquire water and nutrients. Meister et al. (2014) note that the molecular tools to modify root architecture and function are available; however, they still found obstacles in developing improved crop ideotypes. Apart from uncertainty over the optimal combination of shoot and root traits, (Meister et al. 2014) noted that there was a lack of technology for high throughput, non-invasive phenotyping of roots in the field. This applies to both root system architecture and function. The ‘core-break’ method for wheat (Rebetzke et al. 2014; Wasson et al. 2014; White et al. 2015) or the “shovelomics” approach for maize (Trachsel et al. 2011) provide methods for phenotyping root density or architecture in the field, but excavations remain labour intensive and time-consuming. An alternative to excavating the root systems is to measure soil water content as a function of depth and infer root activity from those measurements. Indeed, root access to water deep in the soil profile by roots is positively correlated with higher yields (Ober et al. 2015; Lopes and Reynolds 2010). Soil moisture content can be accurately measured with buried sensors or with probes via access tubes installed in plots, but the cost and effort of instrumenting large experiments is prohibitive, especially when the sensors are left in situ.

The aim of this study was to compare different methods for measuring soil water profiles that could accurately differentiate wheat cultivars, and which could be practically applied to a large number of experimental plots (on the order of several thousand for commercial breeding programmes). In our paper we focus on geophysical approaches to subsurface investigation (Binley et al. 2015), which are usually used for mapping large land areas, and hence may be well suited to monitoring large experiments. However, the signal produced by these measurements depends on the resistivity of the soil, and thus indirectly relates to soil moisture content. Therefore, to provide a reference, we compared these measurements with direct measurements of soil water content with a neutron probe. An earlier paper described how the EMI method was initially tested and developed for root phenotyping (Shanahan et al. 2015). In addition, the extent of soil drying in surface layers was inferred from penetrometer measurements, based on the established relationship between soil matric potential and penetration resistance (Gao et al. 2012, 2016a, b). Both EMI and penetrometer measurements are rapid in comparison with soil coring, taking in the order of a few minutes (no more than 3 min) to collect data from an experimental plot. While soil coring produces direct assessments of rooting profiles, which is immensely valuable (e.g. White et al. 2015), the speed of indirect estimation from soil water profiles is a clear advantage, moreover the two approaches may be used conjunctively to good effect.

Approaches to measure soil drying were compared in a field experiment of 23 different wheat lines over three consecutive years. From these data we concluded that the indirect assessment of root activity determined from soil water profiles is a promising approach that can give reliable and repeatable data that discriminate between wheat lines. However, the most appropriate approach depends on the extent of soil drying, which depends on the season and time of year. We critique different methods of estimating soil water profiles beneath crops where the primary purpose is to make comparisons between genotypes.

## Methods

### The field experiments

#### The field sites

We used two experimental field sites: Warren Field (2013 and 2015) and the neighbouring Broadmead (2014), located in near Woburn, Bedfordshire, UK. The soils are described as a ‘typical alluvial gley soil’ with a texture classification of silty clay loam soil (similar to FAO classification Fluvisol). Both sites are managed by Rothamsted Research and have a long-history under arable agriculture using the mouldboard plough as the primary tillage. In this experiment ‘first wheats’ were sown following at least one year of canola/oilseed rape (*Brassica napus*) break crop. The surface layer (approximately 30 cm) has higher organic matter content and has a lower bulk density than deeper layers. In Warren field wheat roots take up water to a depth of approximately 1 m (Shanahan et al. 2015).

In each year the experimental design was fully randomized complete block in four blocks. Each block contained 23 wheat lines and a fallow plot devoid of vegetation. Plot dimensions were 1.8 m wide and 7.0 m long, and plot ends were cut out to produce a 6 m length for combine harvest. Both sites had the same experimental design but with a different randomization of genotypes. Sowing dates for the 2013 to 2015 harvest years were 01/03/13, 10/10/2013 and 26/9/14, respectively. The 2013 crop was sown late due to poor weather.

In each year, husbandry of the crops followed standard agronomic protocols for the UK, with inputs to ensure adequate nutrition, weed, pest and disease control. No irrigation was applied.

#### Plant material

A panel of 23 winter wheat **(** ***Triticum aestivum*** **L.)** lines were selected to represent the diversity of UK winter wheat germplasm, including current and older varieties, and expression of a range of morphological and physiological traits that could impact root behaviour (Table S1). Some lines are no longer grown commercially (e.g. Robigus), but feature heavily in the pedigrees of many current varieties. A subset of the lines which that showed genotypic differences in root activity was included from a previous study (Ober et al. 2015). A hybrid was also included, as some report that hybrids exhibit greater rooting capacity (Bacon and Beyrouty 1987; Wang et al. 2006). The panel included two sets of isogenic lines to test the effect of *Rht* alleles on root activity, as the literature is not clear on how different *Rht* alleles affect root growth and depth (e.g. Wojciechowski et al. 2009; Miralles et al. 1997). We compared the tall RhtC *(Rht-B1a)* and dwarf *Rht3* (*Rht-B1c*) near isogenic lines, allelic at the Rht-B1 locus, in a Mercia background.

### The instrumentation

#### Electrical resistance tomography (ERT)

ERT has been used to study the variation of soil electrical conductivity in the root zone (Srayeddin and Doussan 2009; Furman et al. 2013). ERT is well suited for use in electrically resistive environments such as dry soil, provided good electrical contact can be achieved between electrodes and soil. The disadvantage for agricultural applications is the requirement for galvanic contact between electrical probes and the soil, resulting in a disturbed soil surface and extensive electrical cabling. The primary purpose of ERT in this work was to calibrate EMI measurements (Shanahan et al. 2015) rather than screen large numbers of genotypes. Calibration of EMI is needed because electromagnetic induction systems return only qualitative values for electrical conductivity because of instrument calibration difficulties (Lavoué et al. 2010). This drawback can be overcome by adjusting EMI data to match the more reliable ERT measurements as described by (Shanahan et al. 2015).

ERT measurements were made at both field sites on two of the four blocks. Fewer blocks were measured with ERT due to combinations of the cost of the arrays and time taken to make the measurements (approx. 1 h/array). These data were later used to assist with analysis of the EMI data (see below). We used four, 96-electrode arrays. Each array had electrodes measuring 0.1 m in length and 0.01 m diameter, inserted into the soil with 0.32 m separation, to give a 30.7 m long array. The arrays remained in position from just after crop emergence until just prior to harvest. A Syscal Pro electrical resistivity meter (Iris Instruments, Orleans, France) was used to measure apparent electrical resistivity, *Ra* over a dipole-dipole electrode configuration (see, for example, Binley 2015). These data were checked for reciprocity of measurements (Parasnis 1988) and then inverted to give a 2-D distribution of soil resistivity using the Occam’s based R2 (version 2.7a) ERT inverse code (Binley 2013). The resistivity was converted to conductivity data (*σ*_ERT_) and on each plot means were taken in the horizontal direction to give a 1-dimensional (1-D) electrical conductivity profile, which could be used to calibrate EMI data (Shanahan et al. 2015) to make it consistent with resistivity profiles determined from ERT. Values of this profile are reported in units of ratio inversion ohm-meters.

#### Electromagnetic inductance

EMI measures apparent electrical conductivity, *σ*_a_, by inductive coupling (e.g., Mester et al. 2011), without the need for contact with the soil surface. EMI is a quick and repeatable method that can be employed at the field and plot scale (Vereecken et al. 2014). The *σ*_a_ measured by EMI represents the weighted average of soil electrical conductivity (*σ)* over a depth range that depends on the separation distance, *s*, between the transmitter and receiver coils, as well as their orientation (McNeill 1980). When *s* is increased the depth of soil contributing to the *σ*_a_ measurement increases (e.g., McNeill 1980; Callegary et al. 2007). Given a set *σ*_a_ measurements obtained with different coil spacing and orientations, a 1-D vertical profile of soil conductivity can be estimated by inverse modelling (Mester et al. 2011; von Hebel et al. 2014).

We used a CMD Mini-Explorer (GF Instruments, Brno, Czech Republic) EMI instrument to make measurements of apparent electrical conductivity at three positions along the centre-line of the experimental plots. The instrument is 1.3 m long, and has a 30 kHz transmitter coil and three receiver coils at different spacing (*s*) from the transmitter (0.32 m, 0.71 m and 1.18 m). The probe can be rotated by 90° (about the long axis) to orientate the coils in a horizontal coplanar (HC) or a vertical coplanar (VC) position. The different coil spacing and orientation allow measurements of *σ*_a_ to be made over six depths within a single position on the plot. The effect of different coil spacing and orientations is to modify the depth of soil that influences the conductivity measurements. The cumulative sensitivity function (McNeill 1980) for vertical coplanar orientation is given by:

1 $$ CS(z)={\left(4{\left(z/s\right)}^2+1\right)}^{1/2}-2\left(z/s\right) $$

and for horizontal coplanar orientation is given by:

2 $$ CS(z)={\left(4{\left(z/s\right)}^2+1\right)}^{-1/2} $$

where *s* is the coil separation and *z* is depth.

Prior to the field campaign we developed a measurement protocol to minimize the effects of instrument drift over time (Shanahan et al. 2015). On each measurement occasion the instrument was allowed to equilibrate to ambient temperatures for at least an hour. A single location at each site, away from the plots being monitored, was established as a “drift base”, where the probe could be returned periodically during each day to assess for any instrument-drift (Corwin and Lesch 2005). These assessments of instrument-drift were then used to adjust all *σ*_a_ measurements. A measurement period of 1.0 s provided *σ*_a_ values with a reasonably low variability (root mean square (RMS) error, typically <2% for most soil conditions). The probe was kept 1.5 m from any metallic items (e.g. electrical cables) to prevent interference. Measurements on all plots were made with one coil orientation, coils were rotated 90° and measurements were repeated.

**Fig. 1:**
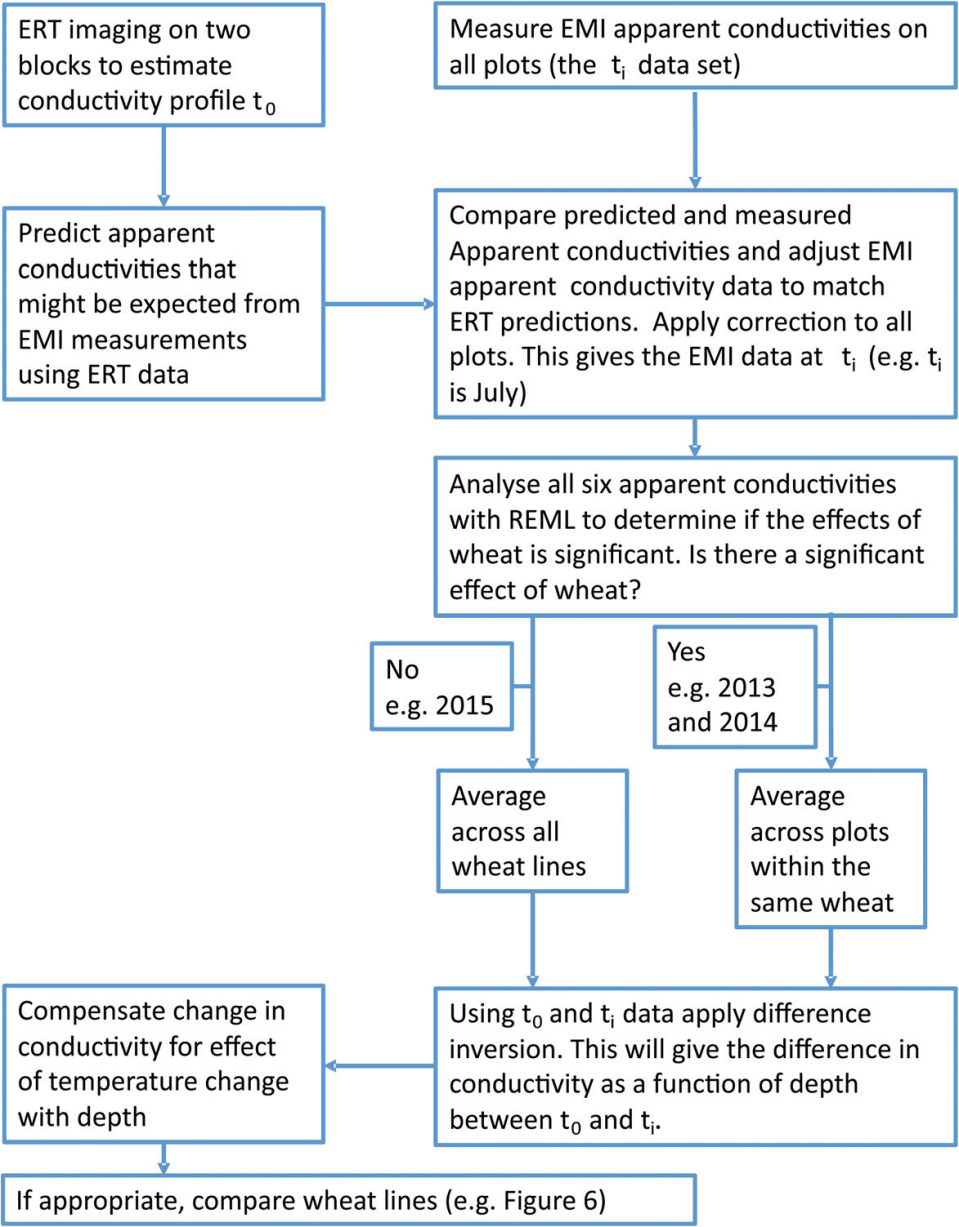
A flow chart to show the steps involved in making and EMI measurement. The flow chart also shows how ERT is used to calibrate EMI apparent conductivity measurements taken in the field

To convert the six measured apparent conductivity (*σ*_*a*_) values into an estimate of how electrical conductivity varies with depth it was necessary to use a refined inversion procedure (as detailed in the Appendix). Shanahan et al. (2015) established the utility of using the change in electrical conductivity with time to infer changes is soil water content. The variable derived from this procedure was termed ‘conductivity from a difference inversion’, and relates to soil drying. The new routine allows the inversion of the difference between two data sets measured at different times with the purpose of fitting the change in conductivity with depth to two sets of *σ*_a_ measurements. Preliminary data showed that soil moisture contents at ≥2 m depth remained stable over the course of a season. Therefore, when estimating the change in conductivity with depth between two time points we used the a priori knowledge that at a depth of 2 m there was no detectible soil drying (and therefore no change in conductivity). The different steps involved in converting apparent conductivity data measured at the plot with an EMI instrument (i.e. the six conductivity values that arise from the different three coil spacings and two orientations) into a conductivity change as a function of depth are summarised in Fig. 1.

#### The penetrometer

The penetrometer is a rod tipped with a steel cone that is manually pushed down through the soil to obtain a relationship between the pressure at the base of the cone (penetrometer resistance) and depth (Bengough and Mullins 1991). For this work we used a recording penetrometer (Solutions for Research, Silsoe, UK) fitted with a 30° cone with 9.45 mm base diameter on a 7.9 mm diameter shaft. The penetrometer has been widely used in soil science, but perhaps more commonly used to detect soil compaction (Campbell and O’Sullivan 1991). For the purpose of this work, the penetrometer was useful because penetrometer resistance is very sensitive to soil water status (Farrell and Greacen 1966; To and Kay 2005; Gao et al. 2012, 2016b). Recently Gao et al. (2016b), proposed the following relationship between penetrometer resistance, *Q*, and other soil properties,

3 $$ Q=\rho {\left({A}^{*}\frac{{\left(F-e\right)}^2}{1+e}{\left({\sigma}_s^p-\psi {\left(\frac{\psi }{\psi_{ae}}\right)}^b\right)}^f\right)}^2 $$

in relatively well-watered field conditions, where *ρ* is the dry bulk density of soil in kN/m^3^, *e* is the void ratio, *σ*_*s*_ is the net stress (kPa), *ψ* is matric potential (kPa) and*ψ*_*ae*_is the matric potential at which air enters a drying soil. F, A*, p, b and *f* are empirical adjustable parameters. Penetrometer resistance can be expected to be closely related to matric potential as well as *σ*_*s*_, which is a function of depth (Gao et al. 2016a).

#### The neutron probe

Neutron moderation is a is well established and widely accepted method to measure soil water content, as described in detail by Gardener et al. (1991). More recently, soil moisture measurements based on dielectric measurements have become common. Sensors based on dielectric measurements, such as time domain reflectance have some important advantages. There is no need for a radioactive source and can be used for a wide range of soil types. In our experience the calibrations between dielectric properties and soil water content are more similar between different soil types than those between the count of thermalized neutrons and soil water content. More recently dielectric probes have been developed that can be inserted into access tubes, allowing measurements of soil water content to depth (e.g. Whalley et al. 2006, 2008). However, the performance of dielectric sensors inserted into access tubes depends on good contact between the tube and the soil. Whalley et al. (2004) found that the calibration of such dielectric probes depended on depth, implying that there was a systematic difference in tube-soil contact with depth from the surface. For this reason, we used the neutron moderation method in this work. Although this approach also uses an access tube to allow measurements of soil water at depth, the instrument samples a much larger volume of soil than dielectric probes, detecting moisture in a radius of 0.15 m in wet soil and 0.5 m in very dry soil (Gardener et al. 1991). In soils that exhibit high shrinkage, Jarvis and Leeds-Harrison (1987) found that neutron probe measurements were affected by shrinkage of soil away from the access tube; however, the extent of the problem is considerably less than that reported for access-tube based dielectric probes (Whalley et al. 2004).

#### Soil characterisation

Soil was collected from Warren field and electrical conductivity was measured in the laboratory on repacked samples in three cores 5.3 cm in diameter and 4 cm long. A four-electrode arrangement, with electrodes connected to a resistance meter (RM4, from Geoscan, Bradford, UK) was used to measure electrical conductivity. Volumetric water content was measured on a separate soil sample using a SM150 dielectric soil moisture sensor (Delta-T Devices Ltd., Cambridge, UK) connected to a logger device. All of the cores were placed in a pressure plate apparatus, and step changes in matric potential between −0.5 and −450 kPa were applied. Before the start of the calibration the soil samples were saturated with 0.01 M NaCl and the water content and electrical conductivity were recorded at equilibrium water contents.

We took undisturbed cores (also 5.3 cm in diameter and 4 cm long) to measure the water release characteristic (Gregory et al. 2010). On these samples we also measured the resistance to a needle penetrometer on the equilibrated samples (Gao et al. 2012).

#### Field measurements

In 2013 we took soil samples to a depth of 1 m, followed by oven drying to estimate water content gravimetrically (Shanahan et al. 2015) on all plots of selected lines (Robigus, Hystar Hybrid, Xi19 and Rht-B1c) and at six time points (24–04-13, 13–05-13, 13–06-13, 20–06-13, 27–06-13, 09–07-13). In 2014 we used a neutron probe to measure soil water content profiles to a depth of 1.45 m on all plots of Battallion, Robigus, Dover, Hybrid Hystar, Rht-B1a and Rht-B1c and at eight time points (19–0-14, 10–03-14, 02–04-14, 09–06-14, 10–04-15, 19–06-14, 26–06-14, 04–07-14 & 17–07-14). In 2015 we used a neutron probe to measure soil water content profiles to a depth of 1.45 m on all plots of the 23 wheat lines and at 12 time points (22–01-15, 23–01-15, 24–02-15, 25–02-15, 18–03-15, 19–03-15, 09–04-15, 10,04,15, 16–04-15, 01–05-15, 08–06-15, 23–06-15). The shift from gravimetric measurements of water content (2013) to the use of the neutron probe (503 Hydro probe, CPN, 5052 Commercial Circle, Concord, CA 94520) reflects the acquisition of the instrument in 2014 and a second instrument in 2015 to allow a greater number of plots to be measured.

At regular intervals during each season ERT and EMI was used to estimate the change in conductivity. In addition, soil temperature profiles were also measured with buried thermistors. The temperature data were used to correct conductivity data to a standard 25 °C to account for differences in temperature. Otherwise differences in either ERT or EMI data could simply reflect temperature differences. The correction rule is a 2% per °C linear increase of the electrical conductivity of the soil with temperature using the following relationship;

4 $$ {\sigma}_{25{}^{\circ}C}={\sigma}_T/\left[1+0.02\left(T - 25\right)\right], $$

with *σ*_*T*_ the electrical conductivity at the temperature *T* (degrees Celsius) and *σ*_25°C_ the electrical conductivity at 25 °C (see for example Michot et al. 2003).

Penetrometer measurements were made during the season until the soil became too strong for the instrument to be used. However, in 2014 there was a more restricted set of penetrometer measurements due to limited staff availability.

At harvest the grain and straw yields from each plot were measured with a plot combine and corrected to a standard 85% dry matter.

### Statistical analysis

All experimental data were analysed with GenStat v16 (www.vsni.co.uk). In each of the experimental years (2013, 2014 and 2015) 23 lines of wheat and a follow plot were set out in a fully randomized complete block in four blocks. A different randomisation scheme was used in each year. The block structure, block/plots, was used for the statistical analyses with a treatment structure of “wheat line” for yield and apparent conductivity measurements and block/plots/depth was used with the treatment structure “wheat line*depth” for the penetrometer. Only conductivity data sets showing a statistically significant treatment effect were inverted to estimate conductivity depth profiles. Penetrometer data was analysed with REML (residual maximum likelihood), but these data required square root transformation to stabilize the variance with spline models to account for the profile with depth. For ease of comparison with other published data we plot penetrometer data on the natural scale and are unable to plot the standard error of differences (SED) which was obtained from the transformed data. In the case of ERT, splines were used to model the profile relationships between conductivity and depth obtained from the inversion procedure after they had been corrected for the effects of temperature. The fitted spline models were compared with REML. Unless stated otherwise we only discuss data as being statistically significant if *P* < 0.001. Yield data was analysed with ANOVA.

## Results and discussion

### Yield

There was significant genotypic variation in grain yields and total above-ground biomass on complementary material (data not shown) within each year when analysed by ANOVA (Fig. S1; *P* < 0.001). All yields in 2013 (sown in spring) were much smaller than in 2014 and 2015. In 2015, yields were consistently between 72 to 92% of the yields in 2014, with the exception of Hobbit and Rht-B1c (dwarf type) where the 2015 yields were greater than in 2014.

### Relationships between soil properties

**Fig. 2:**
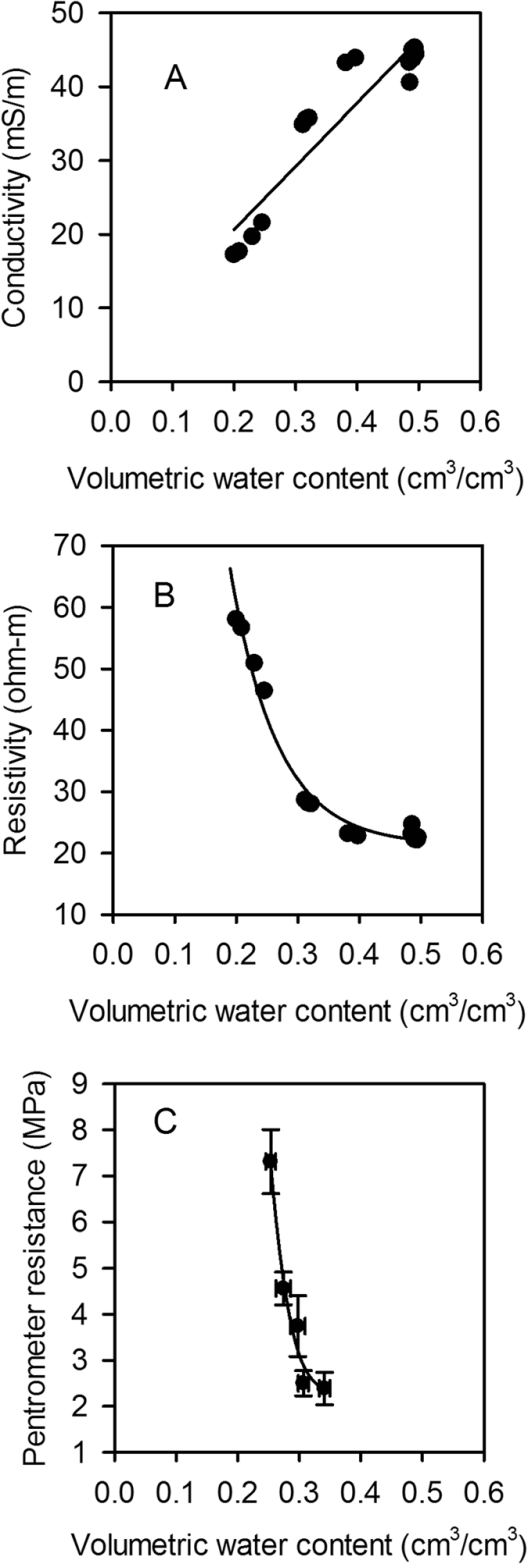
Conductivity (A), resistivity (B) and penetrometer resistance (C) plotted against soil water content. Data are from laboratory measurements of soils collected from Woburn field. Symbols represent the mean ± se (*n* = 3)

**Fig. 3:**
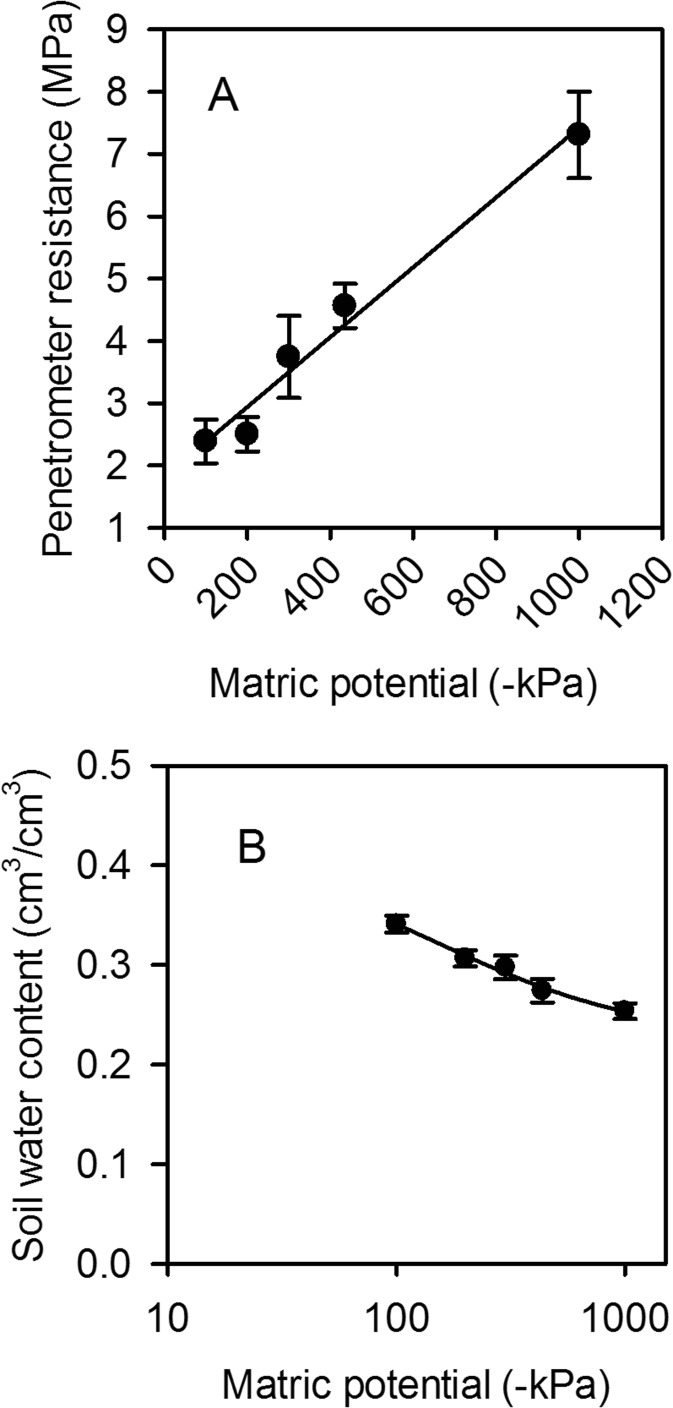
Penetrometer resistance (A) and soil water content (B) plotted against matric potential. Data are from laboratory measurements of soils collected from Woburn field. Symbols represent the mean ± se (*n* = 3)

Laboratory analyses of soils sampled from the trial sites showed that electrical conductivity had a linear relationship with water content over the range considered (Fig. 2). In contrast, there were curvilinear relationships between penetrometer resistance and electrical resistivity and soil water content. In drier soil there will be a much greater change in both penetrometer resistance and electrical resistivity for a given change in water content compared with the same increment in wet soil (Fig. 2). The relationship between penetrometer resistance and matric potential is linear (Fig. 3), assuming *σ*_s_ ≈ 0, which is the case for measurements in the laboratory. The water release curve (the relationship between matric potential and water content) is consistent with those previously reported for the same soil (Fig. 3; Gregory et al. 2010). Although these data (Figs. 2 and 3) are from Warren field, the texture of Broadmead is similar and there is no reason to expect any difference in the conclusions to be drawn from these two soils or indeed other soils with a relatively wide range of clay contents. Taken together, these results show that small changes in soil water content, in dry soil, can result in larger changes in soil matric potential and resistivity; therefore, measurements sensitive to matric potential would be best suited to detect changes in soil drying by roots at low soil water contents.

**Fig. 4:**
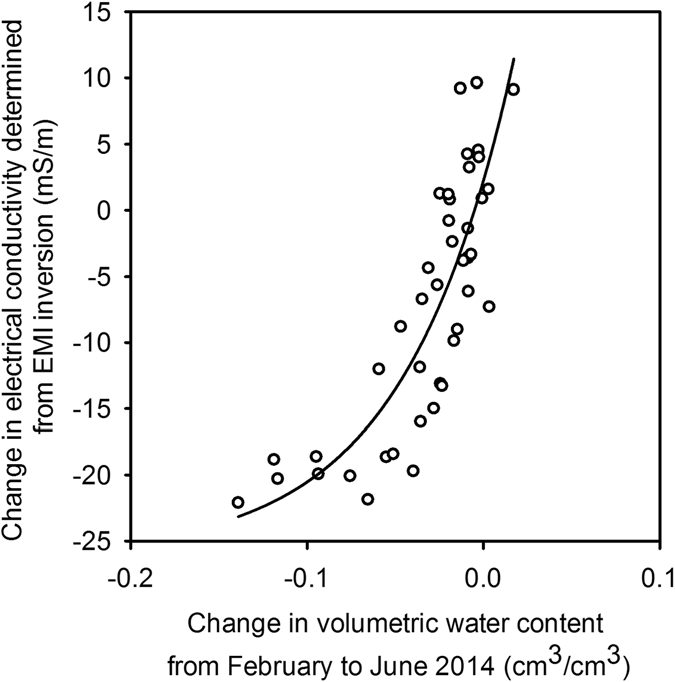
Change in electrical conductivity between February and June 2014, determined from the inversion of EMI data, plotted against the change in water content measured with a neutron probe. These data were obtained from Broadmead field. A negative change in volumetric water content indicates relative soil drying. Symbols represent the individual data points from the different depths measured by the neutron probe and the corresponding conductivity changes determined from the inversion of EMI data

Figure 4 shows the change in electrical conductivity, from Broadmead field in 2014, at various depths, determined from inversion of the EMI data, plotted against the respective change in water content determined with a neutron probe. This relationship is non-linear, but this is mainly due to a relative small data set from the driest soil. This example illustrates that even in dry soil, EMI can detect changes in soil moisture content, but EMI measurements are most sensitive (the steeper section of the curve) in soils that have not dried extensively.

### Soil drying

**Fig. 5:**
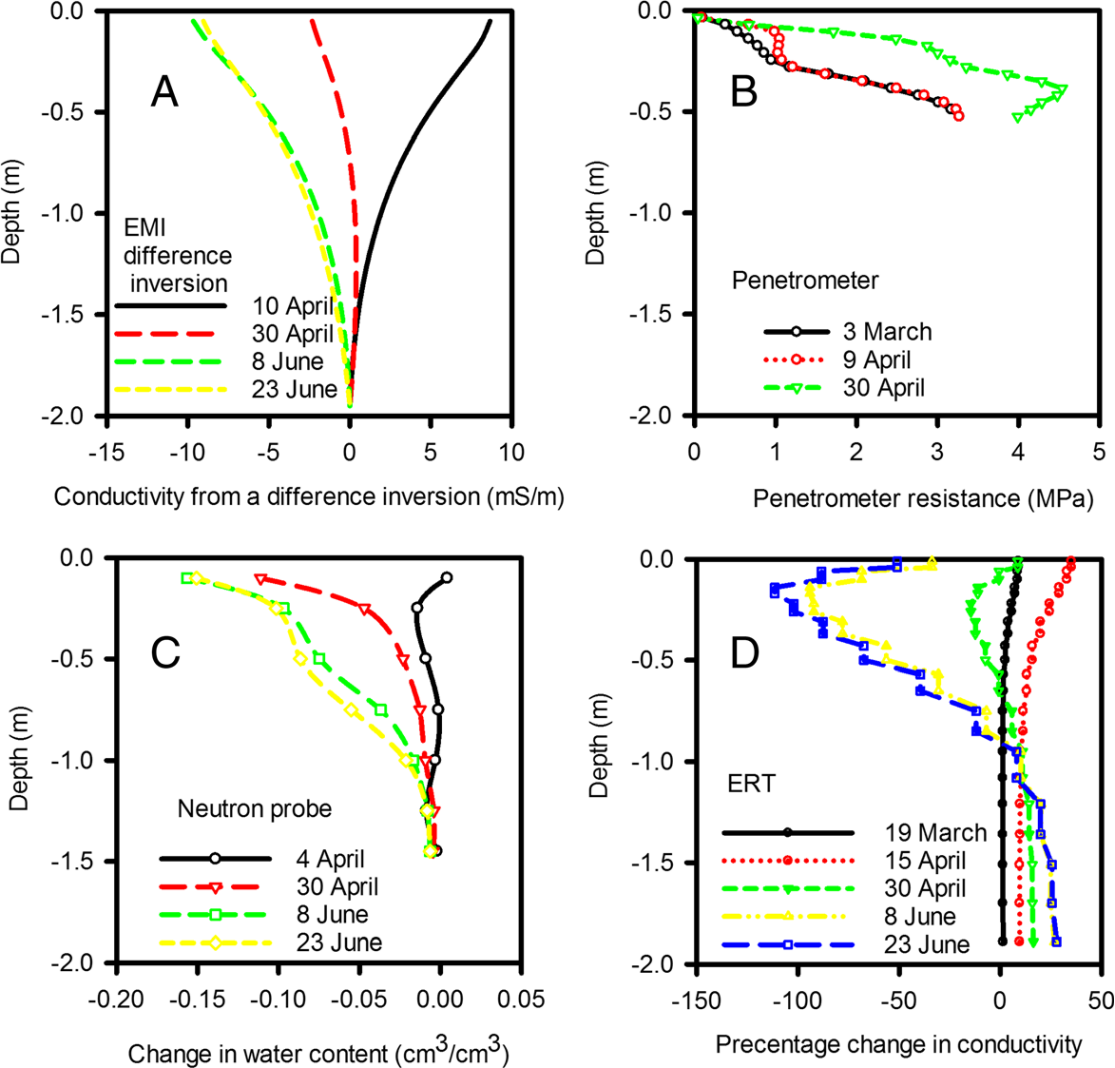
These data were collected in 2015 on Warren field. The effect of temporal patterns in soil drying with depth on data determined from the inversion of ERT (D) and EMI (A) data, penetrometer measurements (B) and neutron probe (C) measurements is shown. EMI and ERT data are obtained from the inversion routine that provides a continuous distribution over depth. We only applied the EMI inversion routine to data sets when the six measured apparent conductivities were significantly different. In 2015 these data were not significantly different and EMI data represent the inversion applied to the average apparent conductivities, taken across all the wheat genotypes. Neutron probe data also showed that there was no significant difference in water profile between wheat genotypes and again the data plotted is the average for all wheats. The penetrometer data are replotted from Gao et al. (2016a). Both Penetrometer and ERT data did show significant genotypic effects (*P* < 0.001), but here we show the average taken across all wheat lines to illustrate the temporal effects. All symbols represent the genotypic grand mean from ANOVA

In all three seasons there was significant soil drying with depth and time, consistent with increased root activity as crop evapotranspiration increased from spring into summer (Fig. S2). In Fig. 5 we show the progressive patterns of inferred soil drying over time for 2015 for each measurement technique. ERT, EMI and neutron probe measurements showed large changes in soil moisture during April, but relatively smaller changes through June. This could reflect rapid depletion of soil moisture by plants earlier in the season compared with later when soils were already relatively dry. Alternatively, as negligible amounts of rainfall were received between 8 and 23 June 2015, that could have masked removal of soil water by roots, the results may indicate that it was more difficult to detect changes in soil moisture content in soils of low moisture content at that time of year. If so, this implies that genotypic effects could be best examined by comparing the relative shifts in patterns of soil characteristics over time between different genotypes rather than relying on the interpretation of differences at one time-point. Similar data were obtained in both 2013 and 2014, except that in 2015 we had a complete set of data from the neutron probe. The greatest difference between 2014 and 2015 was the duration of net soil drying (Fig. S2), which was shorter in 2015 (3 months) than 2014 (5 months). These data are consistent with the differences in rainfall patterns between the two years.

Although the inversion models used to interpret ERT and EMI data provided data to a depth of 2 m, negligible soil drying was observed at depths greater than 1 m during any of the three seasons. In all three seasons we used the penetrometer when the soil was sufficiently weak. In 2015 the manual penetrometer measurements could not be made later than 30 April because as the soil dried it became too strong (Fig. 5). During April 2015, differences in drying of upper soil layers was more apparent from penetrometer measurements than the neutron probe (Fig.5). The large increases in penetrometer resistance associated with relatively small amounts of soil drying is consistent with previously published data (Whalley et al. 2006). However, because *σ*_*s*_ is proportional to depth (Eq. 3; Gao et al. 2016b; Skempton 1987) increases in penetrometer resistance in the near surface layers (<10 cm) are small.

### Differences between wheat lines

**Fig. 6:**
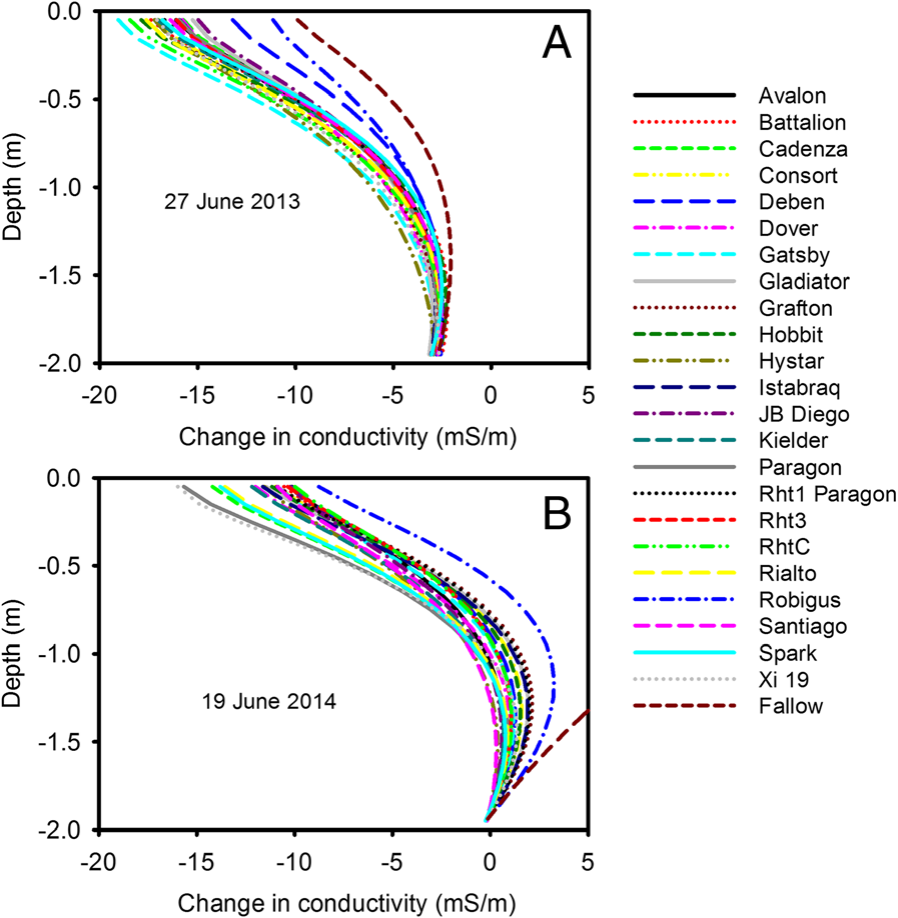
Change in electrical conductivity, determined from inversion of EMI data, as a function of depth for 2013 (A) and 2014 (B). The references dates in 2013 and 2014 were 13–05-13 and 10–03-14 respectively. In 2015 there was no significant effect of wheat line on apparent conductivities measured with EMI, so the data for the individual lines are not shown. The temporal effects of soil drying on the 2015 conductivity profiles are shown in Fig. 5a. In 2013 and 2014 the six measured apparent conductivities were significantly different for the different wheat lines and the inversion routine was applied to each wheat line (see flow chart in Fig. 1)

In 2013 and 2014, residual maximum likelihood (REML) analysis showed significant effects (*P* < 0.001) of soil drying on the six apparent conductivities (corresponding to the three different coil spacing and two orientations), measured with EMI, between the different wheat lines (Fig. 6). However, in 2015 we found no significant effects of genotype on those apparent conductivities measured with EMI. This implied that in 2015 the soil water content profiles of all the wheats were similar, which was supported by neutron probe data (data not shown). Thus in 2013 and 2014 there were sufficient periods of soil drying that allowed differentiation of the genotypes using EMI (Fig. S2), and these apparent conductivity data were inverted relative to a reference date (Fig. 6). In 2013 there was a better separation of the wheat lines which was consistent with the much drier contions in the spring of 2013 (Fig. S2). The shape of the drying curves reflects the general distribution of root biomass in a soil profile, with most root activity occurring in the upper 50 cm (White et al. 2015). Most differences in absolute terms were small, indicating that the EMI method was able to discern relatively subtle genotypic differences in soil moisture extraction. This is important because within elite germplasm in breeding programmes, the genotypic differences in root activity over depth are expected to be small, but potentially important for crop performance (Kirkegaard et al. 2007).

Interestingly, the hybrid wheat line did not show noticeably different patterns of soil drying than most other lines, and in these experiments grain yield was also similar to others (Fig. S1). The effect of different dwarfing alleles also did not result in any distinctive soil drying patterns as detected using EMI. The patterns in Fig. 6 show that soils in plots of Robigus were more conductive (i.e. a smaller reduction in conductivity compared with the reference date) than others, indicating smaller volumes of water extraction. In these experiments, this genotypic effect is probably explained largely by poor resistance of Robigus to yellow rust (*Puccinia striiformis*), as disease pressure was high in 2013 and 2014, and hence a smaller active canopy, rather than an effect of the 1-R/S wheat-rye translocation in Robigus, which has been studied previously in terms of root growth (Sharma et al. 2009). Nevertheless, whatever above-ground processes lead to changes in soil moisture extraction by root systems, any reliable method for field phenotyping roots should be able to detect it.

**Fig. 7:**
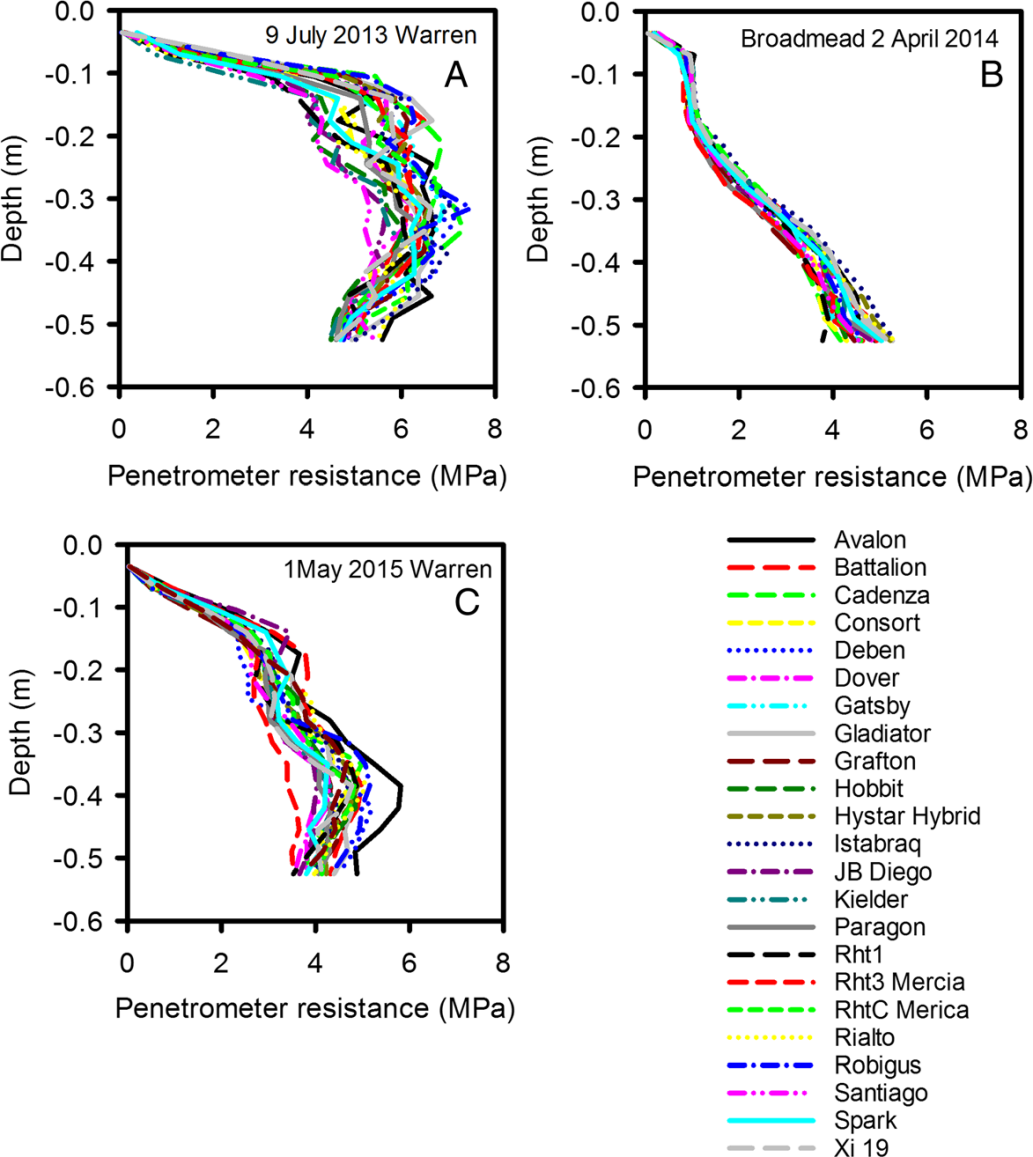
Penetrometer profiles for all three years: 2013 (A), 2014 (B) and 2015 (C). In each case these are the latest date when the soil was still weak enough to push in the penetrometer and obtain a complete data set. REML analysis on the transformed data showed in all of these data sets there was a significance main effect of wheat line (*P* < 0.001) as well as a significant effect of the interaction between wheat line and depth (*P* < 0.001)

**Fig. 8:**
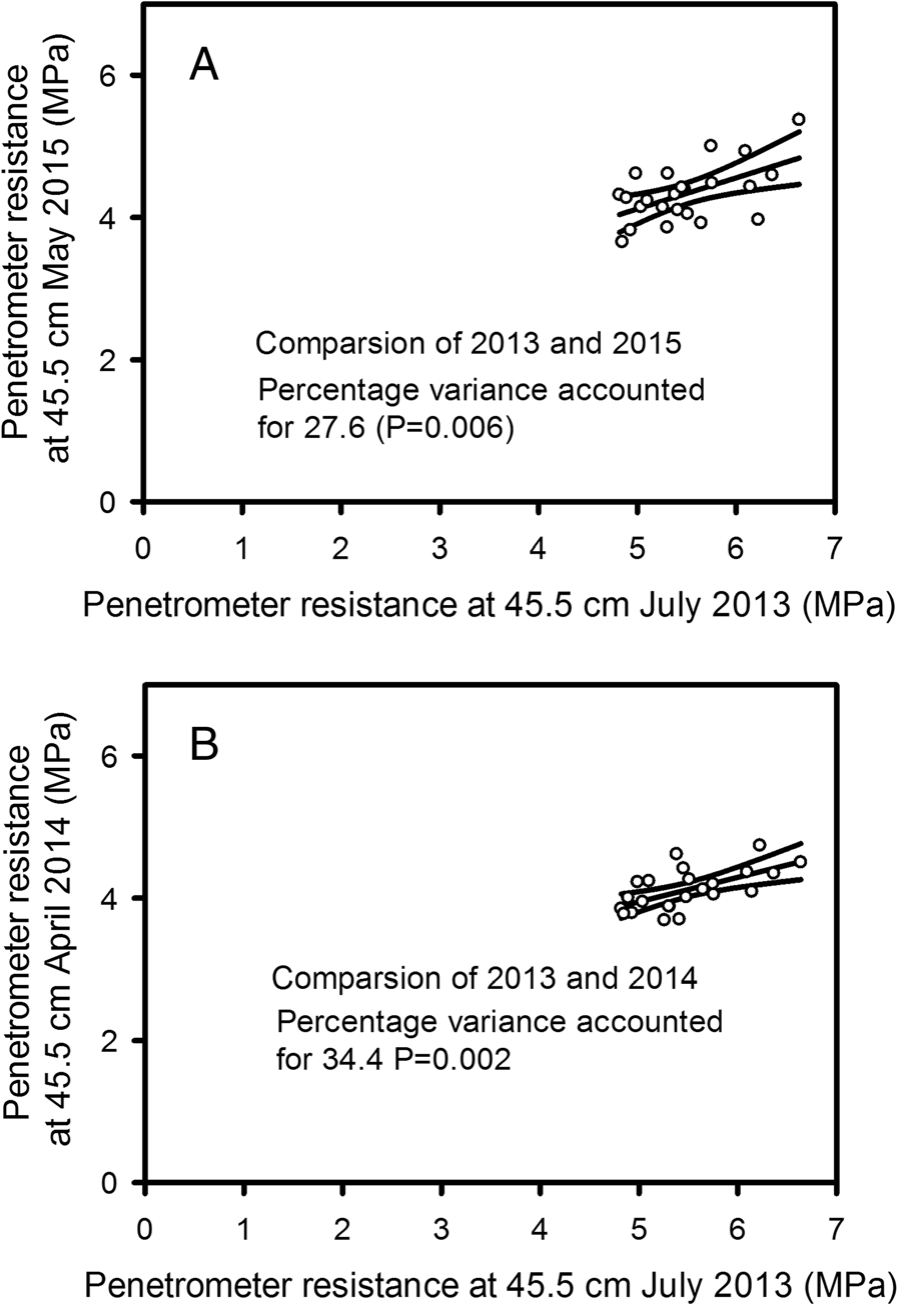
A comparison of penetrometer resistance data at 45.5 cm depth in 2013 with comparable data in 2015 (A) and 2014 (B). Symbols represent the mean for each genotype. The data is supplied as supplementary information

Figure 7 shows the penetrometer data for all three growth seasons. In 2014 the discrimination between the wheat lines was smaller than for either 2013 or 2015. This was because in 2014 the soil profile remained wet until early April when it dried over a relatively short period of time (Fig. S2), and then became too strong for the penetrometer to be used. Penetrometer data were analysed with REML and the interactions between depth and genotype shown in Fig. 7 are all significant at *P* < 0.001. The penetrometer data showed significant genotypic effects in all three years, implying that some wheat lines where more effective at drying soil than others. Furthermore, genotypic rankings for drying at 0.45 m depth were consistent between years (Fig. 8, Table S2). Penetrometer data confirmed the observation made from EMI measurements, that the hybrid wheat did not have a distinctive soil drying pattern. Furthermore, the effect of different dwarfing alleles did not seem to result in distinctive soil drying profiles.

**Fig. 9:**
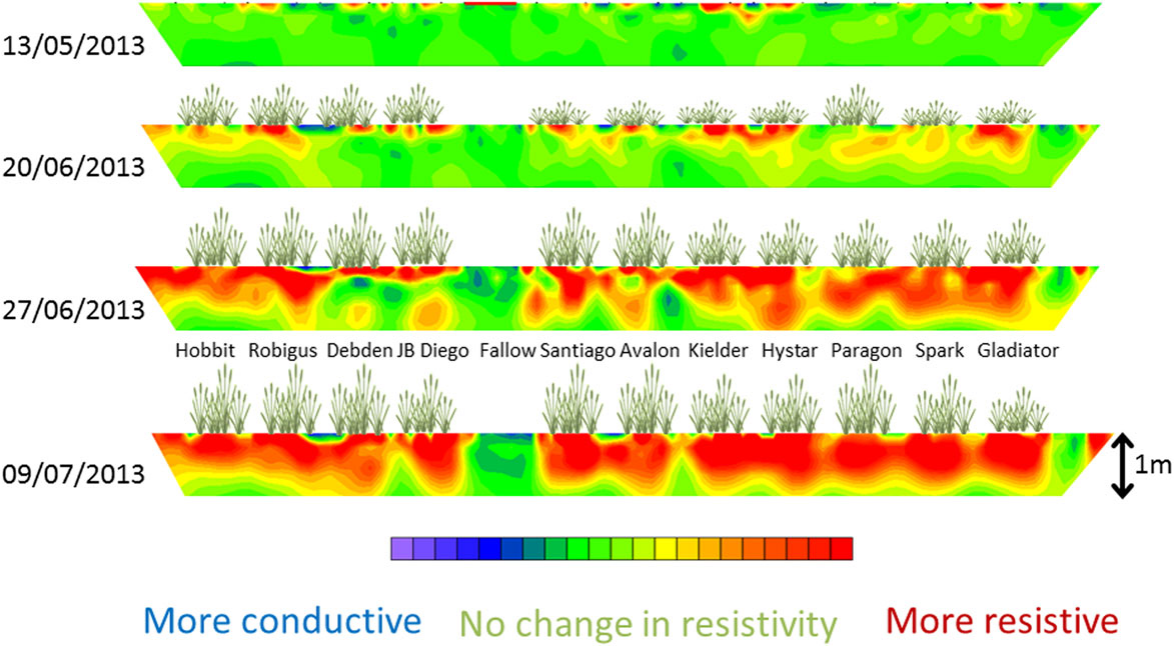
Time lapse images created from inversion of ERT data collected in 2013 from one of the 30 m transects at Warren field. Date format is dd/mm/yyyy. The reference date for the inversion was 23rd April 2013 and the different colours indicate changes in resistivity after that date (red: greater resistivity; green: no change; blue: smaller resistivity). The position of plants in each plot along the ERT transect are indicated, as well as the central fallow plot devoid of plants

In all three years the ERT resistivity data showed significant genotypic effects, but were limited in power because we were able to make measurements in only two of the four blocks. A significant advantage of ERT measurements is that they produce a 2-D map of soil drying (Fig. 9), whereas all the other approaches we have considered are 1-D. ERT is also amenable for use in dry soil, whereas the penetrometer is very sensitive to early soil drying by the roots, but becomes less effective when soils become too strong.

**Fig. 10:**
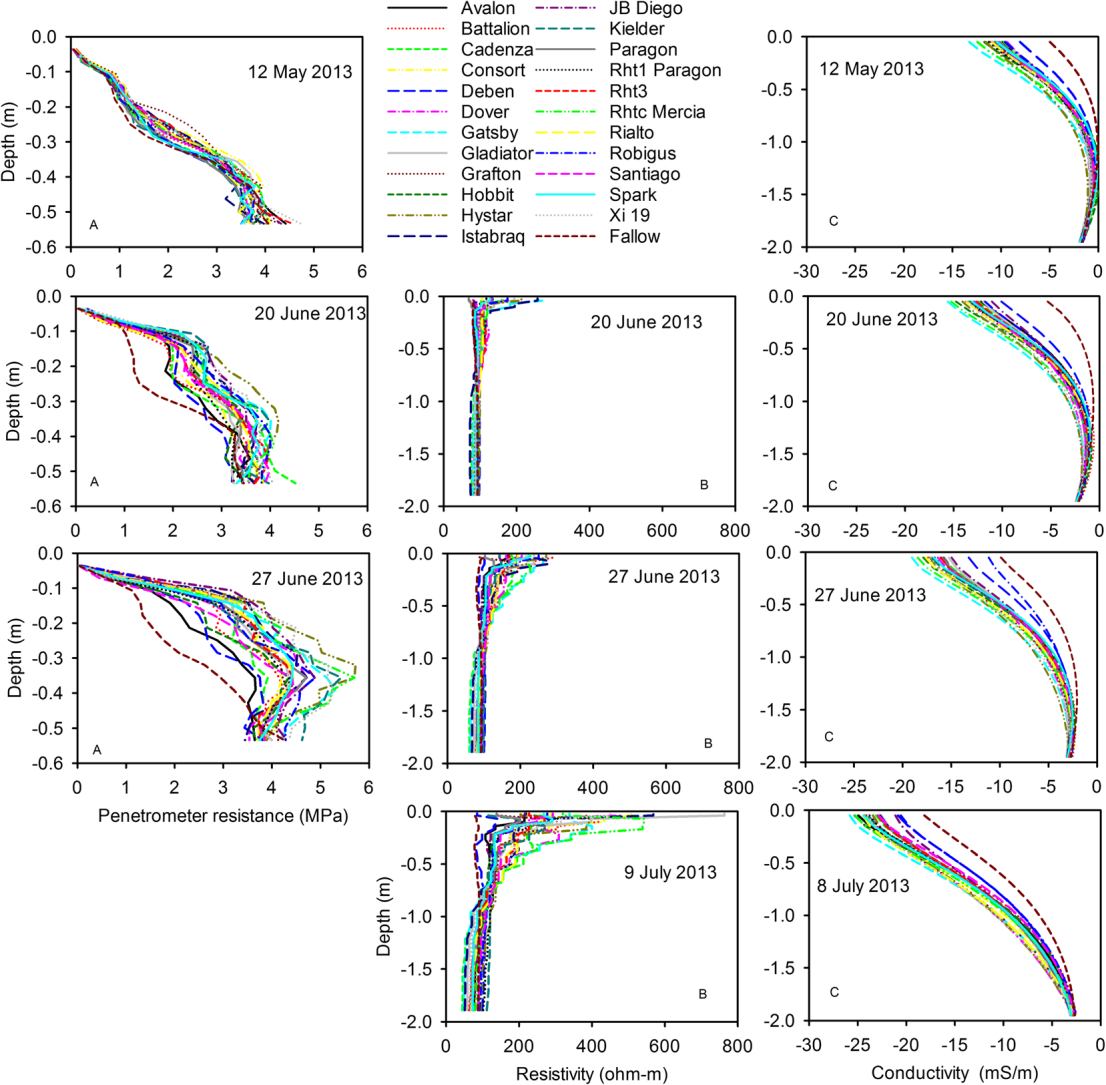
A comparison of depth profiles determined from (B) ERT (resistivity), (C) EMI (conductivity) and (A) penetrometer measurements in 2013. The dates are indicated on the plots. In this panel B a resistivity of 100 Ω-m indicates that there has been no change in resistivity in comparison with the reference date (23rd April); higher values indicate more resistive and drier soil and lower values indicate less resistive and wetter soil

The soil profiles determined with ERT, EMI and penetrometer measurements were compared to look for consistency, and which method provided the best ability to discriminate genotypes (Fig. 10). Data for 2013 are shown because this year had the longest set of comparative data that also showed significant genotype effects with all three methods.

The different sensitivities of ERT, EMI and the penetrometer measurements to soil water content make direct methodological comparisons difficult. Data from the 20 June 2013 (Fig. 10) show penetrometer measurements have the greatest sensitivity to soil drying. On that date there was very little difference between in ERT or EMI data for the different wheat lines, while the penetrometer detected large and significant genotypic differences. On 27 June some wheat lines (Avalon, Battalion, Deben, Cadenza) did not show any detectable soil drying at 50 cm, whereas other lines (Kielder, Xi19, RhtC) appeared to have dried the soil beyond that depth. On the 27 June, penetrometer data and ERT data at a depth of 32 cm gave a consistent genotypic ranking of soil drying (Spearman’s rank correlation; *P* = 0.017). However, the choice of which depth to make the comparison was somewhat arbitrary. At a depth of 50 cm penetrometer data did discriminate well between wheat lines, whereas ERT data did not, at this depth. EMI measurements were adjusted using ERT as a standard measurement (Shanahan et al. 2015), so the agreement between these two methods is inevitably good. By 9 July it was no longer possible to push the penetrometer into the dry soil, which was confirmed by ERT data showing that the soil had dried further from 27 June. Comparison of the EMI and ERT data in July, 2013 (Fig. 10) shows that ERT allowed greater discrimination between wheat lines (i.e. the range of resistivity values at a given depth is large) compared with EMI (where there is a small spread in conductivity data). Both approaches (EMI and ERT) showed statistically significant differences between the wheat lines. The greater sensitivity of ERT (and penetrometer resistance) compared with EMI, was observed more acutely in 2015 where only ERT and penetrometer measurements could detect differences between the wheat lines.

### Critique of the methods

Two basic descriptions of water in soils are the volumetric soil water content and matric potential, which are related to each other by the water release characteristic (Fig. 3). However, the water release characteristic is highly non-linear: small changes in soil water content can correspond to large changes in matric potential. Measurements of matric potential may provide a more sensitive approach to discriminating between the root activity of different wheat lines than measurements of soil water content. In 2015, we found no genotypic differences in soil water content profiles estimated with a neutron probe, nor with EMI-derived conductivity profiles; since conductivity is linearly related to soil water content (Fig. 2). Both neutron probe and EMI measurements showed similar temporal patterns of soil drying with depth in 2015 (Fig. 5). In contrast, in 2015 we did find significant genotypic differences in penetrometer profiles (Fig. 7), which are more closely related to matric potential than water content (Fig. 3; Whalley et al. 2005; Gao et al. 2012, 2016b). The effects of soil depth and soil drying both affect penetrometer resistance, thus the comparisons are simply qualitative. However, the progression of soil drying with depth and over time is clearly visible in the penetrometer data (Fig. 5). The greater sensitivity of penetrometer (and ERT) measurements at discriminating between different wheat lines in 2015 is almost certainly because the soil had been dried to near a residual water content; where small differences in water content (detected by the neutron probe or EMI) correspond to large differences in matric potential, which can be resolved with either penetrometer or ERT measurements. An important finding is that penetrometer measurements revealed a consistent root phenotype across years of soil drying at depth (Fig. 8).

Electrical resistance tomography (ERT) overcomes important limitations of the penetrometer: namely the limited depth and time window before soils harden excessively, preventing measurements. The inversion of ERT data provides soil drying information, in our case, to a depth of 2 m. An advantage of ERT is that it provides a 2-D image of electrical resistivity, which can also be used in the form of a time-lapse image (Fig. 9). As in the case of the penetrometer the relationship between the measured variable in ERT (resistivity) is non-linear with water content (Fig. 2), which makes them both sensitive in dry soils. A limitation of EMI is that it senses electrical conductors and thus is unable to differentiate between very dry (very resistive) and dry (resistive) soil states. Similarly, in dry soil, measurements of water content made with the neutron probes are not accurate enough to estimate the associated larger changes in matric potential (see Fig. 3).

Measurements of soil water that are sensitive to matric potential provided the greatest discrimination between the different wheat genotypes we studied. Indeed, this is entirely consistent with the well understood role of water potential gradients as the driver for root water uptake (Tinker and Nye 2000). In this work we show that penetrometer resistance and ERT measurements are more effective than EMI or the neutron probe at detecting differences in matric potential and hence genotypic differences in root activity. However, in early stages of soil drying when soil water content is relatively high, EMI is also effective in discriminating genotypic differences in root activity.

## Conclusion

This study shows for the first time how EMI, normally used to map soil conductivity over large areas, can be used effectively to quantify genotypic differences in root activity. Our data suggest the indirect phenotyping of roots by measuring soil water is likely to be more effective if the measurement method is related to the matric potential of soil water. Of the methods we have explored in this paper, ERT and penetrometer measurements satisfy this requirement. However, ERT is not amenable to high throughput, and penetrometer measurements are best used in less dry soils and for estimating root activity in upper soil layers. We present evidence that genotypic rankings based on root phenotypes determined from the measurement of soil drying profiles are consistent across years.

### Electronic supplementary material

ESM 1 (DOCX 706 kb)

ESM 2 (DOCX 355 kb)

ESM 3 (DOCX 13 kb)

ESM 4 (DOCX 14 kb)

## Appendix. Inversion of changes in EMI data

Measurements of apparent conductivity (*σ*_a_) can be made in vertical and horizontal coplanar mode, each of which has a different depth sensitivity function, as shown in eq. (1) and (2). The depth of investigation can be altered in each of the two modes by changing the coil spacing *s*. The CMD Mini-Explorer used in this work allows three coil spacings and thus a total of six measurements are possible at a given location. These data may be analysed using an inverse model to determine a profile of electrical conductivity with depth that is consistent with the observed apparent conductivity. Given that we are interested in changes in conductivity (reflecting changes in soil water content), an approach was developed to invert the changes in apparent conductivity between two dates.

We represent the subsurface, at a given time as a one-dimension profile of electrical conductivity, discretised into cells: *σ*_i_ *i* = 1,2,… M, where M is the number of cells. Adopting the cumulative sensitivity function (see eqs. (1) and (2), we can state:

A1 $$ {\sigma}_a=\sum_{i=1}^M{\sigma}_i\left(CS\left({z}_{i-1}\right)-CS\left({z}_i\right)\right) $$

where z_i_ is the depth to the centre of cell i and z_0_ = 0.

If we consider the change in apparent conductivity (*Δσ*_a_) between two dates, for a given coil spacing and coil orientation, we can write:

A2 $$ \Delta {\sigma}_a=\sum_{i=1}^M{\varDelta \sigma}_i\left(CS\left({z}_{i-1}\right)-CS\left({z}_i\right)\right) $$

where *Δσ*_i_ is the change in conductivity in cell i.

We wish to determine the profile of values ∆*σ*_i_ that minimises the difference between the six observed differences in apparent conductivity.

Simplifying notation for what follows, we can write our observed changes in apparent conductivity as *d*_*i*_, *i* = 1,2,..,6; the set of parameters (change in conductivity for each cell) that we wish to determine as *m*_*i*_, *i* = 1,2,… M; and a forward model operator (eq. (A2)) as *f*_*i*_, *i* = 1,2,..,6. We can then write an objective function to be minimised, in a weighted least squares sense, as:

A3 $$ \Psi =\sum_{i=1}^6{\left(\frac{\Big({d}_i-{f}_i\left(\boldsymbol{m}\right)}{\varepsilon_i}\right)}^2 $$

where *ε*_*i*_ is error in observation i.

To avoid an undetermined inverse problem and to ensure a smooth profile of changes in conductivity (consistent with expected behaviour) we can revise our objective function to incorporate a spatial regularisation term:

A4 $$ \Psi =\sum_{i=1}^6{\left(\frac{\Big({d}_i-{f}_i\left(\boldsymbol{m}\right)}{\varepsilon_i}\right)}^2+\alpha \sum_{i=1}^{M-1}{\left({m}_{i+1}-{m}_i\right)}^2 $$

where α is a scalar that weights the regularisation against the data misfit.

Minimising the objective function in eq. (A4) leads to the set of equations:

A5 $$ \left({\boldsymbol{J}}^{\boldsymbol{T}}{\boldsymbol{W}}^{\boldsymbol{T}}\boldsymbol{W}\boldsymbol{J}+\alpha \boldsymbol{R}\right)\mathbf{m}={\mathbf{J}}^{\mathbf{T}}\left(\mathbf{d}-\mathbf{f}\right) $$

that is solved to determine the parameters **m**, and where: **J** is the jacobian, given by J_i,j_ $=\frac{\partial \Delta {\sigma}_{a,i}}{\partial \Delta {\sigma}_j}$, *i* = 1,2,…,6 and *j* = 1,2,…,M; W_i_ = (1/ε_i_), **R** is a roughness matrix that is defined in such a way to represent the right had side of eq. (A4).

Using eq. (A2) we can write the jacobian terms in eq. (A5) as:

A6 $$ \frac{\partial \Delta {\sigma}_{a,i}}{\partial \Delta {\sigma}_j}=CS\left({z}_{j-1}\right)-CS\left({z}_j\right) $$

where the cumulative sensitivity function *CS* is selected to represent the appropriate coil separation and orientation for measurement i.
