# Supplementary material for: Methods to estimate changes in soil water for phenotyping root activity in the field
Source: Plant Soil. 2017 Jan 12;415(1):407–22. doi: 10.1007/s11104-016-3161-1 (PMC6979655; doi:10.1007/s11104-016-3161-1)
Supplement: Supplementary file 1 — (DOCX 706 kb) [file 11104_2016_3161_MOESM1_ESM.docx]

Figure S1. Wheat yields for all three seasons of this study. The 2013 yields are much smaller because this crop was spring sown due to poor weather in the autumn of 2012 and very late establishment of that year’s crop. Bars represent the mean of four replications. The SED values for 2013, 2014 and 2015 were 0.381, 0.541 and 0.895 Tonnes/ha (66 df) respectively with P<0.001 in all cases.
