# Supplementary material for: Methods to estimate changes in soil water for phenotyping root activity in the field
Source: Plant Soil. 2017 Jan 12;415(1):407–22. doi: 10.1007/s11104-016-3161-1 (PMC6979655; doi:10.1007/s11104-016-3161-1)
Supplement: Supplementary file 2 — (DOCX 355 kb) [file 11104_2016_3161_MOESM2_ESM.docx]

Figure S2. In the top panel, the rainfall (blue bars) and potential soil moisture deficit data (PSMD) for the duration of this study are shown. Data are from the weather station at Rothamsted’s Woburn experimental farm. In the bottom panel the temporal patterns of soil drying measured with a neutron probe in 2014 and 2015 are shown (no neutron probe data was available in 2013). The data are the mean data taken across all the wheat lines. The reference date is 19 February in 2014 and 23 January in 2015, when soil profiles were assumed to be fully recharged with water.
