# Supplementary material for: Methods to estimate changes in soil water for phenotyping root activity in the field
Source: Plant Soil. 2017 Jan 12;415(1):407–22. doi: 10.1007/s11104-016-3161-1 (PMC6979655; doi:10.1007/s11104-016-3161-1)
Supplement: Supplementary file 3 — (DOCX 13 kb) [file 11104_2016_3161_MOESM3_ESM.docx]

| ID | Variety | Wheat Breeder  (<http://wheatpedigree.net/>) |
| --- | --- | --- |
| 1 | Battalion | Monsanto |
| 2 | Consort | Plant Breeding International Cambridge Ltd |
| 3 | Gladiator | Plant Breeding International Cambridge Ltd |
| 4 | Istabraq | Nickerson (UK) Ltd |
| 5 | Robigus | CPB Twyford Ltd. |
| 6 | Xi19 | Advanta Seeds B.V., PO Box 1, 4410 AA Rilland,Netherlands |
| 7 | Rhtc Mercia | A gift to Rothamsted from Prof Snape (John Innes Centre) |
| 8 | Rht1 Paragon | A gift to Rothamsted from Dr. Griffiths (John Innes Centre) |
| 9 | Rht3 Mercia | gift to Rothamsted from Prof Snape (John Innes Centre) |
| 10 | Deben | Nickerson (UK) Ltd |
| 11 | Dover | CPB Twyford Ltd. |
| 12 | Gatsby | Advanta Seeds UK Ltd |
| 13 | Rialto | Plant Breeding International Cambridge Ltd |
| 14 | Spark | Nickerson Seeds |
| 15 | Hobbit | Plant Breeding International Cambridge Ltd |
| 16 | Avalon | Plant Breeding International Cambridge Ltd |
| 17 | Cadenza | Plant Breeding International Cambridge Ltd |
| 18 | Paragon | Plant Breeding International Cambridge Ltd |
| 19 | Santiago | KWS UK Ltd |
| 20 | Grafton | CPB Twyford Ltd |
| 21 | Kielder | KWS UK Ltd |
| 22 | JB Diego | Josef Breun, 91074 Herzogenaurach, Germany |
| 23 | Hystar hybrid | Saaten Union |
|  |  |  |

Table S1. Wheat lines used in this study
