# Supplementary material for: Methods to estimate changes in soil water for phenotyping root activity in the field
Source: Plant Soil. 2017 Jan 12;415(1):407–22. doi: 10.1007/s11104-016-3161-1 (PMC6979655; doi:10.1007/s11104-016-3161-1)
Supplement: Supplementary file 4 — (DOCX 14 kb) [file 11104_2016_3161_MOESM4_ESM.docx]

Table S2. The data plotted in Fig. 8

| wheat | 2013 at 45.5 (July) | 2014 at 45.5 cm (April) | 2015 at 45.5 (May) |
| --- | --- | --- | --- |
| Avalon | 6639 | 4506 | 5375 |
| Battalion | 4986 | 4228 | 4618 |
| Cadenza | 5258 | 3689 | 4142 |
| Consort | 5410 | 3702 | 4105 |
| Deben | 5749 | 4201 | 5006 |
| Dover | 4931 | 3792 | 3821 |
| Gatsby | 5309 | 3884 | 4619 |
| Gladiator | 6366 | 4349 | 4597 |
| Grafton | 5757 | 4054 | 4483 |
| Hobbit | 4820 | 3852 | 4320 |
| Hystar hybrid | 5382 | 4620 | 4326 |
| Istabraq | 6226 | 4743 | 3969 |
| JB Diego | 5038 | 3949 | 4149 |
| Kielder | 5101 | 4240 | 4237 |
| Paragon | 4891 | 4004 | 4274 |
| Rht1 Paragon | 4849 | 3778 | 3654 |
| Rht3 Mercia | 5480 | 4015 | 4404 |
| Rhtc Mercia | 6145 | 4093 | 4433 |
| Rialto | 6095 | 4370 | 4932 |
| Robigus | 5648 | 4125 | 3921 |
| Santiago | 5304 | 3885 | 3860 |
| Spark | 5510 | 4265 | 4051 |
| Xi19 | 5451 | 4418 | 4420 |

Regression analysis output from GenStat

Regression analysis

Response variate: %2014_at_45_5_cm

Fitted terms: Constant, %2013_at_45_5

Summary of analysis

Source d.f. s.s. m.s. v.r. F pr.

Regression 1 699569. 699569. 12.54 0.002

Residual 21 1171561. 55789.

Total 22 1871130. 85051.

Percentage variance accounted for 34.4

Standard error of observations is estimated to be 236.

*Message: the following units have large standardized residuals.*

Unit Response Residual

11 4620. 2.33

*Message: the following units have high leverage.*

Unit Response Leverage

1 4506. 0.267

Estimates of parameters

Parameter estimate s.e. t(21) t pr.

Constant 2224. 538. 4.13 <.001

%2013_at_45_5 0.3452 0.0975 3.54 0.002

36 "Simple Linear Regression"
 37 MODEL %2015_at_45_5
 38 TERMS %2013_at_45_5
 39 FIT [PRINT=model,summary,estimates; CONSTANT=estimate; FPROB=yes; TPROB=yes] %2013_at_45_5
Regression analysis

Response variate: %2015_at_45_5

Fitted terms: Constant, %2013_at_45_5

Summary of analysis

Source d.f. s.s. m.s. v.r. F pr.

Regression 1 1125082. 1125082. 9.40 0.006

Residual 21 2514011. 119715.

Total 22 3639094. 165413.

Percentage variance accounted for 27.6

Standard error of observations is estimated to be 346.

*Message: the following units have large standardized residuals.*

Unit Response Residual

12 3969. -2.14

*Message: the following units have high leverage.*

Unit Response Leverage

1 5375. 0.267

Estimates of parameters

Parameter estimate s.e. t(21) t pr.

Constant 1930. 788. 2.45 0.023

%2013_at_45_5 0.438 0.143 3.07 0.006
